# Substance use among healthcare professionals: A cross-sectional study in Kenya

**DOI:** 10.1371/journal.pgph.0003863

**Published:** 2025-03-24

**Authors:** Jasmit Shah, Cynthia Smith, Cyprian Mostert, Linda Khakali, Zul Merali, Sayed K. Ali

**Affiliations:** 1 Brain and Mind Institute, Aga Khan University, Nairobi,; 2 Department of Internal Medicine, Aga Khan University, Nairobi; University of Washington, UNITED STATES OF AMERICA

## Abstract

Substance use among healthcare professionals, including alcohol, tobacco, marijuana, benzodiazepines etc., is a rising concern. The immensity of substance use is not yet clear, though recently, studies conducted on medical students and physician trainees show that 10% to 15% of all healthcare professionals misuse drugs at some point in their career, and that 6% to 8% of physicians have a substance use disorder. Substance use has a direct impact on relationships, physical health, and job performance, and is associated with depression, burnout, and decreased career satisfaction. The aim of this study was to shed light on the prevalence of substance use in healthcare providers in Kenya. We carried out a cross-sectional survey study between May 2023 and August 2023. The participants were healthcare providers in Kenya, including medical doctors and trainees, nurses, and allied healthcare workers, who could be reached via email or WhatsApp. The standardized English questionnaire consisted of questions based on demographics and behavior characteristics, as well as substance use. A total of 1768 healthcare providers across Kenya participated and were included in the analysis. The median age of participants was 36.0 years (IQR: 31.0, 44.0), 45.1% being males, 67.1% were married, 46.9% were from public institution, and 34.0% were doctors. The prevalence of ever using a substance was 51.7%. Of the participants who reported ever using any substance, the most commonly used substance was alcohol (93.7%), followed by cannabis (28.9%) and tobacco products (27.6%). Age, race, marital status, religion, education, institution type, type of healthcare provider, and income were associated with substance use (p<0.05). In conclusion, about half of this convenience sample of healthcare providers in Kenya reported ever using one of these substances. Given the potential negative impact of harmful substance use on health service delivery and on providers’ mental and physical health, institutions and government should devote resources and create constructive interventions to further understand and address the scope of these issues in Kenyan healthcare providers.

## Background

Globally, the number of people suffering from substance use disorders (SUDs) has increased by 45% over the last decade. In sub-Saharan Africa (SSA), the burden of SUDs is projected to increase upto 130% by 2050 [1]. According to the World Health Organization (WHO), cannabis remains the most widely used illicit substance in the African region, followed by amphetamine type stimulants [2]. Additionally, the Global Status Report on Alcohol and Health in 2018 reported that Africa had the highest global prevalence of heavy episodic drinking among the individuals aged 15 – 24 years [3]. In Kenya over 10% of the population aged 15 – 65 years have an alcohol use disorder, and one in every six individual aged 15-65 years use at least one substance of abuse. Data from the 2022 national survey on the status of drugs and substance use in Kenya revealed that alcohol was the most widely used substance in the population, and though the prevalence of substance use was on a downward trend, the prevalence of cannabis use had almost doubled in the last five years [1,4].

Many factor place HCPs at a higher risk of SUD and include: extended duration of medical training, extreme competition, stress, excessive workload, burnout, fear of failure, compassion fatigue and frequent contact with illness and death, [5–7] Additionally, substance use, and SUDs are often difficult to detect in HCPs, and this is thought to be due to their high baseline functioning, skills training, and tendencies to deny or minimize any drug related issues [7,8]. HCPs are also less likely to seek help due to embarrassment, worry for their reputation, fear of stigma, fear of personal and work-related consequences [8]. Substance use has been strongly associated with depression, burnout, suicidal ideation, decreased quality of life, and decreased career satisfaction. These consequences may translate to poor patient care and have the potential to exacerbate existing healthcare service discrepancies, particularly where there is a shortage of HCPs especially in LMICs [9,10].

A study conducted at a Nigerian tertiary hospital found that 4.1% Nigerian doctors were hazardous alcohol users, while 12.0% were moderate users [6]. A more recent Nigerian study reported that psychological distress in HCPs was significantly associated with harmful alcohol use, and this psychological distress was significantly associated with 10-20 years’ experience in healthcare. This study also found a higher rate of alcohol use in doctors compared to nurses [11]. Similarly, a study in South Africa found 11% of HCPs to be current smokers and 22% to have alcohol use problems, and that male HCPs were 13 times more likely to smoke than female HCPs [12]. Additionally, in Tanzania, burnout in HCPs was significantly associated with tobacco use [13]. In Kenya, lifetime substance use in HCPs was found to be higher than the general population. For all substances included in the study except hallucinogens and sedatives, men were reported to have higher substance use rates than women [14]. Another study in Kenya conducted at the beginning of COVID-19 found a high percentage (43.9%) of reported harmful patterns of alcohol use in healthcare workers. Factors associated with harmful alcohol use included being doctors and specialists, male, unmarried or with 11–20 years’ experience in the healthcare field [15]. From the current literature, there seem to be emerging similarities in the trends and factors associated with substance use in HCPs in Africa. However, there remains a clear scarcity of research on substance use among HCPs, especially in Kenya. As of 2022 only 2 studies of this nature had been conducted [5], highlighting the dire need for an update on the prevalence and patterns of substance use in Kenyan HCPs.

HCPs play a primary role in modelling lifestyle and in educating and promoting health [16]. Their substance use could have far-reaching consequences on the general population [14]. It is therefore crucial to understand their patterns of substance use. As evidenced by the scarcity of data available in Africa concerning substance use among HCPs, there seems to be a great mismatch between the burden of substance use and the attention it gets. This study aims to shed light on the prevalence of substance use in healthcare providers in Kenya across different cadres, and the factors associated with their substance use.

## Methods

We carried out a cross-sectional survey study between 1^st^ May 2023 and 31^st^ August 2023. The participants were healthcare providers in Kenya, including medical doctors and trainees, nurses, and allied healthcare workers, who could be reached via email or WhatsApp. The doctor category included consultants who are providers who have completed their training and licensed to practice independently, residents who are pursuing a Masters in Medicine, fellows who are pursuing a sub-specialty training post a Masters in Medicine and house/medical officers who have completed their medical school (Bachelors in Medicine) and 1 year of internship. The questionnaire was in English, as it was assumed that the participants, having undergone medical or healthcare training in Kenya or practicing in Kenya, would possess the necessary fluency of English to understand and respond to the survey. The survey consisted of questions based on demographics, behavioral characteristics and substance use. The survey used in this study was developed based on previous literature and discussion with the research team. Furthermore, the survey was built using the Alcohol, Smoking, and Substance Involvement Screening Test (ASSIST) by WHO and a questionnaire for baseline survey on alcohol and drug abuse by the National Authority for the Campaign Against Alcohol and Drug Abuse (NACADA) in Kenya [17,18]. The tools used in this study assessed for only the non-medical use of substances. Non-medical use refers to the consumption of substances for reasons other than prescribed medical purposes, such as recreational use or misuse. To reduce potential bias of self-reported data, confidentiality of participants and privacy of their responses was prioritized. Participants were recruited through a combination of professional networks, institutional contacts, and WhatsApp; commonly used by HCPs in Kenya. Emails lists/listservs were obtained from the institutions and professional groups. Participation messages were disseminated by a few champions identified across different regions through existing WhatsApp groups and networks, including those managed by professional associations, institutional departments, and peer groups. All recruitment methods were adhered to ethical guidelines, ensuring that participation was voluntary, and no personal identifiers were obtained. Data were collected through an online survey on REDCap [19]. Online written consent was obtained from all the participants. The study link was disseminated through emails and WhatsApp. Approval for this study was obtained from the Institutional Scientific and Ethics Review Committee (ISERC) at the Aga Khan University, Nairobi (Ref: 2023/ISERC-11(v2)), and National Commission for Science Technology and Innovation (NACOSTI) (Ref:584850). Participants were allowed to withdraw from the study at any time without any consequences as this was a voluntary participation and no compensation was received.

Continuous data were analyzed using medians and interquartile ranges (IQRs) whereas categorical data were analyzed as frequencies and percentages. The non-parametric Kruskal–Wallis test was used to compare the continuous variables and Fisher’s exact test or Chi-square test was used to compare the categorical variables between group associations. Data analysis was performed using SPSS statistical software V. 20.0 (IBM, Armonk, NY, USA) and R software (R-4.3.1). The basemap shapefile for Kenya was obtained from openAfrica (https://open.africa/dataset/kenya-counties-shapefile). The significance level was set at α = 0.05, and all tests were two-tailed.

## Results

### Overall characteristics of participants

A total of 1,768 HCPs participated in the study, 34.0% were doctors, 34.5% were nurses and 31.4% were allied health staff. Among doctors, consultants represented 41.7%, residents 23.5%, fellows 3.2%, house/medical officers 28.5%, and medical students 3.2%. The study included participants from various counties in Kenya, with Nairobi having the highest representation of 24.5%. Other notable counties included Tharaka-Nithi at 12.0%, Kiambu at 7.3%, and Uasin Gishu at 7.4%. Some counties had minimal representation, such as West Pokot, Makueni, and Mandera. The mean age of participants was 37.9 years (SD=9.0), with the minimum age of 21 years and a maximum age of 71 years. The gender distribution indicated that 45.1% (797) were male, 54.6% (965) were female. The racial composition predominantly comprised of African participants at 95.2%, followed by Asian at 3.4%. Among the participants, 67.1% were married and 30.6% were single. The majority of 85.4% were Christian, followed by 10.6% Muslim and 1.5% Hindu. Within education level, 39.1% of participants had a bachelor’s degree, 35.1% held a diploma (least education), 24.9% possessed a master’s degree and 0.9% held a doctorate (most educated). In terms of income, 41.3% of participants reported earning less than 100,000 Kenya Shillings, while 25.3% earned between 100,000 and 200,000 Kenya Shillings, and only 8.1% reported incomes greater than 300,000 Kenya Shillings. The healthcare workers were affiliated with different types of institutions, the 46.9% majority coming from public, 38.3% coming from private and 13.4% from faith-based institutions.

### Prevalence of substance use in Kenyan HCPs

**Fig 1:**
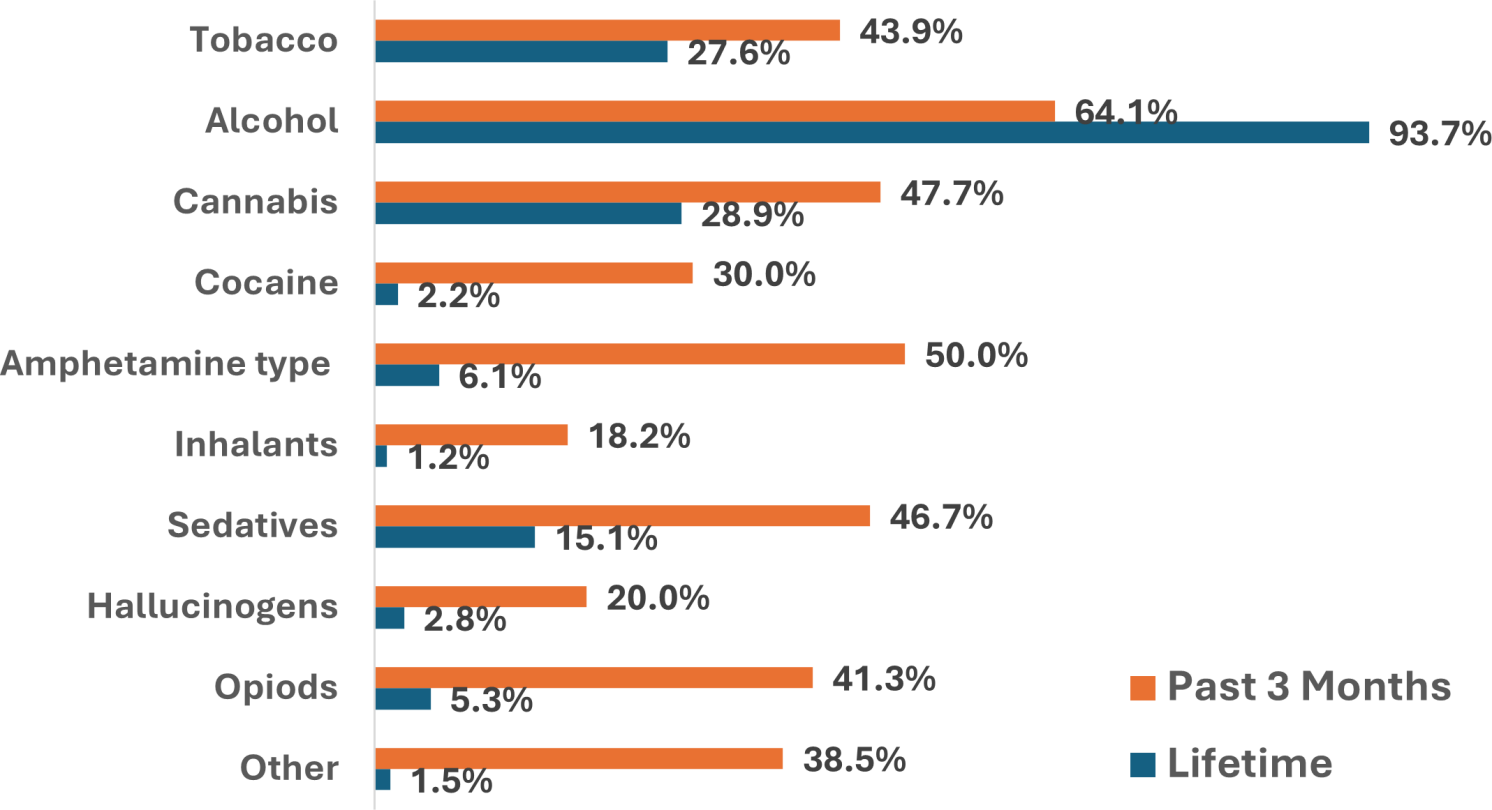
Lifetime use and past 3-month use of substances. *The “past 3 months” percentages are based on the subset of those reporting lifetime use.

**Fig 2:**
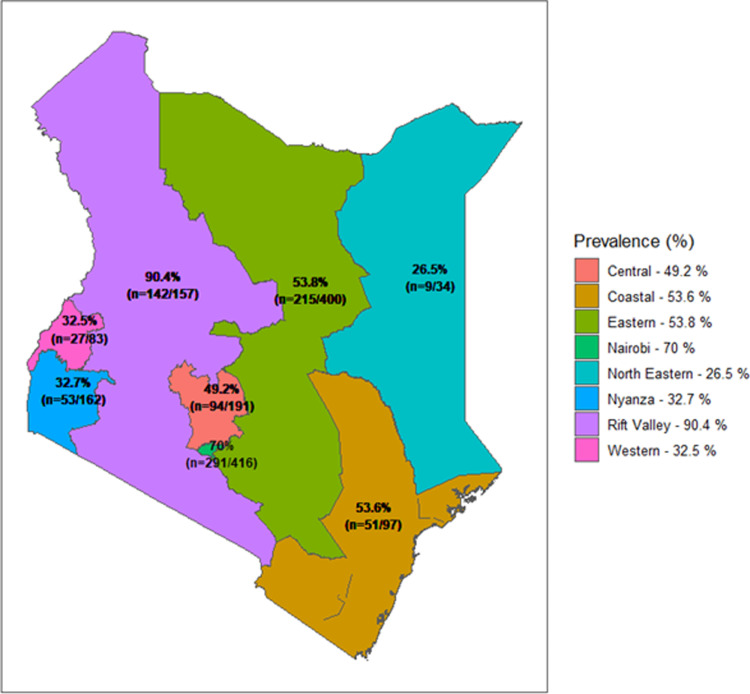
Showing prevalence of substance use in the different regions of Kenya. *Base shapefile for Kenya was downloaded from https://open.africa/dataset/kenya-counties-shapefile. The terms and conditions are further available at https://open.africa/about/terms-and-conditions.

This study found that 51.7% of the HCPs had used a substance in their lifetime. Fig 1 shows the prevalence of substance use in HCPs, majorly alcohol and tobacco, which are legal and widely available. Alcohol was the most used substance, with 93.7% of those having ever used, whereas cannabis was the second most used substance, with tobacco products following. Based on the region, lifetime use was highest in the Rift Valley province at 90.4% (n=142/157), followed by Nairobi province at 70.0% (n=291/416) as shown in Fig 2.

### Results from ASSIST questionnaire

Fig 1 shows the comparison between lifetime use of any substance and use within the past 3 months. “Lifetime use” refers to any use of a substance at least once over the course of a person’s life, regardless of the frequency or recency. Participants who reported “lifetime use” were then asked whether they had used the substance in the past 3 months. As shown in Fig 1, alcohol was the most commonly reported substance for lifetime use, with 93.7% of participants indicating they had ever used it. Among those who reported lifetime use of alcohol, 64.1% indicated that they had used it within the past 3 months.

Tables 1 and 2 summarize additional findings. Participants who had used substances in their lifetime reported that drugs from the ‘other’ category caused the most concern among friends and family members, and failure to do what was expected. Tobacco products and alcoholic beverages had similar percentages for causing concern among friends and family members. However, tobacco products were reported as the most daily used substance, followed by opioids, then cannabis.

**Table 1:** Results summarizing from the ASSIST questionnaire for the past 3 months.

	In the past 3 months					
	How often have you had a strong desire or urge to use?		How often has your use led to health, social, legal or financial problems?		How often have you failed to do what was normally expected of you because of your use of	
	Never	Have had	Never	Have had	Never	Have had
Tobacco products (cigarettes, chewing tobacco, cigars, etc.)	34.9% (n=38)	65.1% (n=71)	78.0% (n=85)	22.0% (n=24)		
Alcoholic beverages (beer, wine, spirits, etc.)	38.6% (n=210)	61.4% (n=334)	75.0% (n=408)	25.0% (n=1360	81.4% (n=443)	18.6% (n=101)
Cannabis (marijuana, pot, grass, hash, etc.)	29.0% (n=36)	71.0% (n=88)	89.5% (n=111)	10.5% (n=13)	91.1% (n=113)	8.9%
Cocaine (coke, crack, etc.)	33.3% (n=2)	66.7% (n=4)	83.3% (n=5)	16.7% (n=1)	83.3% (n=5)	16.7% (n=11)
Amphetamine type stimulants (speed, diet pills, ecstasy, etc.)	32.1% (n=9)	67.9% (n=19)	85.7% (n=24)	14.3% (n=4)	82.1% (n=23)	17.9% (n=1)
Inhalants (nitrous, glue, petrol, paint thinner, etc.)	50.0% (n=1)	50.0% (n=1)	100.0% (n=2)	0.0% (n=0)	100.0% (n=2)	0.0% (n=0)
Sedatives or Sleeping Pills (Valium, Serepax, Rohypnol, etc.)	31.3% (n=20)	68.8% (n=44)	78.1% (n=50)	21.9% (n=14)	84.4% (n=54)	15.6% (n=10)
Hallucinogens (LSD, acid, mushrooms, PCP, Special K, etc.)	40.0% (n=2)	60.0% (n=3)	80.0% (n=4)	20.0% (n=1)	100.0% (n=5)	0.0% (n=0)
Opioids (heroin, morphine, methadone, codeine, etc.)	15.8% (n=3)	84.2% (n=16)	73.7% (n=14)	26.3% (n=5)	84.2% (n=16)	15.8% (n=3)
Other - specify: (E.g. Khat)	60.0% (n=3)	40.0% (n=2)	80.0% (n=4)	20.0% (n=1)	80.0% (n=4)	20.0% (n=1)

**Table 2:** Results summarizing from the ASSIST questionnaire for the past 3 months.

	Has a friend or relative or anyone else EVER expressed concern about your use of		Have you EVER tried and failed to control, cut down or stop using	
	No	Yes	No	Yes
Tobacco products (cigarettes, chewing tobacco, cigars, etc.)	84.1% (n=212)	15.9% (n=40)	82.5% (n=208)	17.5% (n=44)
Alcoholic beverages (beer, wine, spirits, etc.)	84.7% (n=725)	15.3% (n=131)	86.8% (n=743)	13.2% (n=113)
Cannabis (marijuana, pot, grass, hash, etc.)	92.0% (n=243)	8.0% (n=21)	93.2% (n=246)	6.8% (n=18)
Cocaine (coke, crack, etc.)	90.0% (n=18)	10.0% (n=2)	90.0% (n=18)	10.0% (n=2)
Amphetamine type stimulants (speed, diet pills, ecstasy, etc.)	96.4% (n=54)	3.6% (n=2)	94.6% (n=53)	5.4% (n=3)
Inhalants (nitrous, glue, petrol, paint thinner, etc.)	100.0% (n=11)	0.0% (n=0)	100.0% (n=11)	0.0% (n=0)
Sedatives or Sleeping Pills (Valium, Serepax, Rohypnol, etc.)	89.1% (n=123)	10.9% (n=15)	89.1% (n=123)	10.9% (n=15)
Hallucinogens (LSD, acid, mushrooms, PCP, Special K, etc.)	96.2% (n=25)	3.8% (n=1)	96.2% (n=25)	3.8% (n=1)
Opioids (heroin, morphine, methadone, codeine, etc.)	87.5% (n=42)	12.5% (n=6)	81.3% (n=39)	18.8% (n=9)
Other - specify: (E.g. Khat)	71.4% (n=10)	28.6% (n=4)	92.9% (n=13)	7.1% (n=1)

Participants also reported difficulties controlling, cutting down, or stopping use, most frequently with opioids, tobacco products and alcoholic beverages. For substances used within the past 3 months (Table 2), the substances reported to cause the strongest desires or urges to use, were opioids and cannabis, and sedatives or sleeping pills. Amphetamines and cocaine were reported with similar percentages. The substances most reported to cause health, social, legal and financial problems were opioids and alcoholic beverages. The ‘other’ drugs category, where some participants (6 of 14) mentioned khat – a stimulant plant that is legal in Kenya [20], was most reported to cause participants to fail to do what was normally expected of them due to their use.

Very few participants (0.5%) who had ever used a substance in their lifetime reported injecting drugs for non-medical use in the past 3 months and only 1.0% reported injecting drugs more than 3 months before. Of the participants who used tobacco products, 40.4% also used e-cigarettes. Regarding alcohol consumption, 5.2% of participants reported having at least one drink on 5 or more days per week, while 25.9% reported having at least one alcoholic beverage 1-4 days per week. Additionally, 32.6% of participants reported having a parent/guardian who used drugs and/or substances of abuse, and 36.1% had a friend who used drugs and/or substances of abuse.

### Factors associated with use in substance users compared to non-substance users

Substance use among healthcare workers was associated with various demographic factors such as age, marital status, race, religion, education, type of institution they work in, cadre of healthcare worker, specialty, and income (Table 3). Healthcare workers who were slightly younger were more likely to use substances, and the median age between the two groups was statistically significant (34 years vs 37 years, H (1) =19.462, p<0.001). Asians were more likely to use substances than Africans (*X*^2^ (2) = 10.23, p=0.005). Being single was associated to substance use (*X*^2^ (2) = 31.49, p<0.001). Christians were more likely to use substances (*X*^2^ (3) = 45.42, p<0.001). Participants with higher levels of education, that is master’s and above, were more likely to use substances (*X*^2^ (3) = 21.77, p<0.001). Participants from private and faith-based institutions were more likely to use substances than participants from public institutions (*X*^2^ (2) = 7.57, p=0.023). Doctors and allied health staff were more likely than nurses to use substances (*X*^2^ (2) = 22.55, p<0.001). The specialties of surgery and internal medicine were significantly associated with substance use (*X*^2^ (1) = 4.17, p=0.049 and *X*^2^ (1) = 5.15, p=0.023). Finally, health care workers who reported earning higher salaries, were more likely to use substances (*X*^2^ (3) = 9.14, p=0.027).

**Table 3:** Differences among characteristics of participants based on substance use.

		NO USE		YES USE		P Value
Age (years) (n=1345)		37.0 [32.0, 45.0]		34.0 [30.0, 42.0]		<0.001
Gender	Male	371	46.5%	426	53.5%	0.165
	Female	482	49.9%	483	50.1%	
Race	African	825	49.1%	854	50.9%	0.005
	Asian	17	28.3%	43	71.7%	
	Other	11	44.0%	14	56.0%	
Marital Status	Single	213	39.4%	327	60.6%	<0.001
	Married	625	52.8%	558	47.2%	
	Other	12	30.8%	27	69.2%	
Religion	Christian	705	46.9%	798	53.1%	<0.001
	Muslim	126	67.4%	61	32.6%	
	Hindu	10	38.5%	16	61.5%	
	Others	8	18.2%	36	81.8%	
Education	Diploma (Least Educated)	329	53.2%	290	46.8%	<0.001
	Bachelors	333	48.3%	356	51.7%	
	Masters	190	43.2%	250	56.8%	
	Doctorate (Most Educated)	1	6.3%	15	93.8%	
Type of Institution	Public (Government)	424	51.2%	404	48.8%	0.023
	Private	318	47.0%	358	53.0%	
	Faith Based	98	41.5%	138	58.5%	
Type of HCW	Allied Health Staff	262	47.3%	292	52.7%	<0.001
	Nurse	339	55.8%	269	44.2%	
	Doctor	253	42.2%	346	57.8%	
Type of Doctor	Consultant	99	39.8%	150	60.2%	0.356
	Trainee	152	43.7%	196	56.3%	
Specialty	Anesthesiology	11	31.4%	24	68.6%	0.159
	Emergency Medicine	18	62.1%	11	37.9%	0.052
	Family Medicine	25	36.8%	43	63.2%	0.242
	Internal Medicine	119	49.2%	123	50.8%	0.023
	Obstetrics and Gynecology	17	47.2%	19	52.8%	0.729
	Pathology	13	39.4%	20	60.6%	0.718
	Pediatrics	9	34.6%	17	65.4%	0.420
	Radiology	5	41.7%	7	58.3%	1.000
	Surgery	18	31.0%	40	69.0%	0.049
Type of Surgery	Neurosurgery	4	36.4%	7	63.6%	0.772
	Cardiothoracic surgery	2	33.3%	4	66.7%	1.000
	Pediatric surgery	1	20.0%	4	80.0%	1.000
	Plastic and Reconstructive surgery	0	0.0%	5	100.0%	0.309
	Urology	2	28.6%	5	71.4%	1.000
	Vascular surgery	2	28.6%	5	71.4%	1.000
	Breast and endocrine surgery	3	42.9%	4	57.1%	0.664
	Surgical Gastroenterology	6	42.9%	8	57.1%	0.322
	Hepatopancreaticobiliary surgery	0	0.0%	4	100.0%	0.299
	Surgical oncology	3	50.0%	3	50.0%	0.359
	Bariatric surgery	3	50.0%	3	50.0%	0.359
	Trauma and critical care surgery	3	25.0%	9	75.0%	0.735
Income (Kenya Shillings)	Less than 100,0000	329	45.4%	396	54.6%	0.027
	100,000 - 200,000	198	36.4%	346	63.6%	
	200,000 - 300,000	41	34.7%	77	65.3%	
	Greater than 300,000	50	35.0%	93	65.0%	

Furthermore, looking at the top four lifetime use of substances (tobacco, alcohol, cannabis and sedatives), the use of all four substances was higher in doctors as compared to nurses and allied staff. Tobacco use by doctors was 51.0% as compared to 32.9% by allied staff and 16.1% by nurses. Alcohol use by doctors was 37.5% as compared to 32.8% by allied staff and 29.7% by nurses. Cannabis use by doctors was 58.4% as compared to 29.4% by allied staff and 12.1%. Sedatives use by doctors was 44.2% as compared to 28.3% by allied staff and 27.5% by nurses.

## Discussion

Substance use among HCPs in Kenya is of major concern. While use does not necessitate abuse, it can still lead to impairment, affect the quality of care and be detrimental to patient safety [8]. Indeed, compared to many other vocations, the direct consequences of substance use on service delivery in HCPs can have severe effects on the lives of many. This is particular significant in the context of Kenya, where there is already a significant shortage of HCPs, and a single HCP can be responsible for the health outcomes of many patients [10,15].

In this diverse convenience sample of HCPs, 51.7% reported having used a substance in their lifetime, which is slightly lower than the general Kenyan population aged between 15-65 years, where lifetime use was reported to be 57% [4]. The 51.7% figure for lifetime substance use refers to the proportion of HCPs in the convenience sample who reported ever using any substance at least once during their life, whether it was alcohol, tobacco, cannabis, opioids, or other substances.

A majority of our participants had tried alcohol in their lifetime, and it was the most frequently used substance in the past three months. Previous findings in Kenyan HCPs similarly found the highest lifetime use of any substance was for alcohol at 35.8%, significantly lower than our own findings of 93.7% [14]. However, only 5.2% of our participants reported consuming at least one alcoholic beverage 5 or more days a week, and 25.9% reported using 1-4 times a week. Over half of the participants who used alcohol reported a strong desire or urge to use in the past 3 months, and a quarter reported their use had led to health, social, legal and financial problems. Importantly, 18.6% of HCPs who used substances in this study reported failing to do what was expected of them due to their alcohol use in the past 3 months. This harmful pattern of use, which may affect HCP performance, is concerning as it may also directly affect patients seeking healthcare services. Doctors and nurses require optimal cognitive performance due to the nature of their jobs, and the effects of alcohol on cognition are dependent on patterns of use. While moderate consumption has been suggested to be beneficial over time [21], binge drinking, hangovers, as well as intoxication and withdrawal may still affect HCP performance and have lasting detrimental effects on cognition and decision making [22,23]. Our study did not measure patterns of alcohol consumption nor units consumed, which can be important for deciphering these effects on HCP performance, and an important consideration for the design of future substance use studies.

Interestingly, cannabis was the second most reported substance for lifetime use in our study at 28.9%, which is significantly higher than the previous findings of 9.3% in Kenyan HCPs [14]. While cannabis use in the general population of Kenya is said to have almost doubled in the last five years, overall lifetime use among those aged 15-65 year remains much lower than our study population, at 3% [24]. A recent meta-analysis done on studies from various continents, found a 37% lifetime use of cannabis in MD’s and medical students, higher than the 25% lifetime use among 15–64-year-olds in the general European population. Work stress was suggested as a potential cause for this difference and may offer a possible explanation to our findings as well. The daily use of cannabis from this meta-analysis was at 0.1% in physicians [25], much lower than our own daily use findings of 5.4%.

Tobacco products had the third highest percentage for lifetime use at 27.6%, similar to previous findings in Kenyan HCPs of 23.5% [14]. Among our participants who used substances, almost half reported using tobacco products in the past three months and these were also the most used daily substance. Both studies on Kenyan HCPs found higher lifetime usage of tobacco than in the general population in Kenya, where lifetime use was 15% [4]. Quitting smoking has immediate health benefits, and therefore raising awareness on the benefits of stopping smoking is crucial. HCPs can play a vital role in counseling their patients on smoking cessation. However, evidence suggests that doctors who smoked or were former smokers were less likely to counsel their patients on quitting smoking. These findings also translated to other behavioral practices of doctors, such as nutrition counselling from doctors who followed healthy diets [26]. This suggests that the lifestyle choices of HCPs may indirectly impact behavioral choices of their patients, though this needs to be investigated further in Kenya.

Our results for lifetime use of opioids was higher than previous findings in Kenyan HCPs at 5.3%, compared to previous findings of 3.9%. Our study also found higher lifetime use in sedatives or sleeping pills than previous findings. However, our findings for lifetime use of cocaine, inhalants and hallucinogens were lower, and amphetamine type stimulants also had a marginally lower lifetime use in our study compared to previous findings[14]. For all of these substances, there is evidence suggesting lingering effects on cognitive abilities which may affect HCP performance, even following a period of abstinence [7,27]. Therefore, understanding patterns of use and raising awareness of the lasting effects of substances of abuse is crucial in Kenyan healthcare settings. Among our substance using cohort, opioids were reported to cause the strongest desire or urge to use, and led to the most health, social, legal or financial problems in the last 3 months. Additionally, participants who used opioids were most likely to report difficulties in controlling, cutting down or stopping use, which remains a concerning fact.

In this study, being unmarried was correlated with substance use, mirroring other research findings [5,15,25]. Being unmarried is thought to result in a lack of social support and social isolation, which are both risk factors for substance use [15]. Research has suggested that the change in social role associated with marriage has a causal deterrent effect on psychoactive substance use [28]. It would be useful for future research to determine if these reasons for decreased substance use translate into the Kenyan context as well, in order to better understand patterns of substance use in our population.

Though there was a slightly higher percentage of female substance users in this study, the difference in prevalence between the genders was not statistically significant, contrasting previous findings. The male gender (sex at birth) is strongly associated with substance use in both HCPs and the general population [4,5,12,14–16,24,25]. Substance use affects genders differently, both physically and in terms of roles, norms and consequences; and therefore, affects the pattern and prevalence of substance use, generally translating into fewer female substance users [14,29]. More investigation is required to not only replicate but also determine why there may be no difference in substance use prevalence between the genders in Kenyan HCPs.

This study had a relatively young population of HCPs, so the age difference between substance users and non-users was not large. Despite this, there was statistical significance that suggested younger HCPs are more likely to use substances. This may be compared to findings from previous research [16,25,30,31] which show that younger HCPs may be more likely to be unmarried, possibly leading to the same risk factors described above such as social isolation. Younger HCPs may be new to their fast-paced, highly stressful work environments and may perhaps turn to substances to cope; however, this theory requires further examination in our context.

Doctors and allied health staff were more likely to use substances than nurses. This is consistent with previous findings in Kenya and Nigeria, where it was suggested that nursing is a majority female-dominated profession, while doctors were more likely to be males, and hence a likely association with substance use [11,15]. Furthermore, it has been suggested that nurses in Kenya have strong social support systems that may prevent unhealthy coping using substances, which perhaps sheds light on our findings [15]. Another factor may be the prescription privileges doctors have that nurses do not, which may lead to higher accessibility to controlled substances for doctors and potentially explain part of the use prevalence difference in the cadres. Self-prescription of medicines is a unique factor that may lead to substance use in HCPs. Hartnett and colleagues found that anesthetists and surgeons were more likely to self-prescribe potentially addictive medications, underestimating their risk for addiction due to their familiarity with the drugs. General practitioners were also more likely to self-prescribe, and this was suggested to be because of their perceived stigma of attending a colleague [32]. In our findings, the specialties of surgery and internal medicine were also linked to a higher prevalence of substance use. The intense nature of surgery and potentially being in life-or-death situations daily, may lead to increased substance use to help cope with work related stress [9,33]. Additionally, potentially high workloads and long hours for internal medicine physicians in Kenya may propagate unhealthy coping habits, but this theory merits further investigation.

HCPs from private or faith-based institutions were more likely to use substances than participants from public institutions. The reasons for this are largely unknown, however there is evidence of faith based hospitals having poor organizational structures [34] which could possibly lead to less regulation of substances of abuse. Regardless, further investigation into higher rates of substance use in HCPs in faith-based organizations is needed. One possible explanation for the higher rates of substance use in private facilities may be due to higher income brackets for HCPs in these facilities.. Our findings are similar to findings from a study done in Brazil, where physicians and nurses with higher incomes were more likely to use alcohol [16]. In addition, a multi-country analysis of 55 low income and middle-income countries also found a clear positive association between increasing socioeconomic status and current drinking. The association between increasing wealth quantiles and current drinking was stronger in females than males [35]. These results are supported by a systematic review on the prevalence of tobacco use in healthcare workers, where it was found that in low and middle income countries (LMICs), there was a positive association between smoking and higher socioeconomic status particularly in women [36]. Higher socioeconomic status may facilitate easier access to substances due to increased affordability and doctors tend to earn more as compared to the nurses or the allied health staff. Additionally, higher income may be linked to higher educational attainment, which can provide access to more resources. This study, like others [14,35], found that HCPs with higher levels of education were also more likely to use substances. One possibility is that higher educational attainment in HCPs is associated with a better job position and higher income, leading to higher affordability and accessibility of substances. More in-depth examination of the associations between socioeconomic status of HCPs and substance use in Kenya is required, as well as research on reasons for different prevalences in substance use in different healthcare institutions.

### Future directions for substance use research

There is limited data on the patterns and prevalence of substance use in Kenyan HCPs, and additional research is needed to identify avenues for intervention. Future research should aim to include quantity measures as well as when substances are being used, to better understand potential effects of this use on HCP performance. Further research into age and the effects of marital status are also required. Substance use patterns, have been shown to be affected by subjective feelings of loneliness [37]. Therefore, to further explore the relationship between marriage, social support and substance use, it would be useful for future substance use studies to consider factors such as subjective feelings of loneliness, or lack of social support in married and unmarried HCPs.

Additionally, though our findings did not show any differences in prevalence between the male and female gender, other evidence suggests there are differences in types of substances used as well as patterns of use, between the genders. Reduction of gender-blind substance use research is not only important for the advancement of gender equity, but can also improve efficiency, accuracy and reproducibility [29], and further shed light on potential opportunities for intervention.

Furthermore, in one meta-analysis, lifetime use of cannabis in physicians and the general population was seen to vary significantly among continents due to differing laws, cultural and religious beliefs [25], and these factors should be taken into account in future comparative studies.

Substance use is more likely to be a coping mechanism in HCPs, and hospital work culture may promote and maintain substance use [13,38]. Therefore, studies into hospital work culture in Kenya would be informative, including investigating workplace stigma and perceived support, as well measuring levels of workload and burnout in HCPs.

Finally, in this study, the “Other” category had the highest percentages for failure to do what was expected and causing concern among friends and family of HCPs. A number of HCPs reporting use of “Other” substances listed Khat and miraa. Khat/miraa is an amphetamine-like stimulant substance that is legal and widely available in Kenya. Its use has however been linked to several adverse health effects as well as deficits in several cognitive domains [20,39]. It would be insightful to determine the prevalence and effects of khat use in Kenyan HCPs.

## Conclusion

In conclusion, this study found a concerningly high lifetime and previous 3 months use of many substances of abuse when compared to previous studies in Kenyan HCPs, as well as the general population. Tobacco products were the most daily used substances, and opioids caused the strongest desire or urge to use, as well as health, social, legal and financial problems and failure to cut down or stop using. Factors associated to substance use in Kenyan HCPs included: being unmarried, having higher socioeconomic status, being a doctor or allied health staff, being a surgeon or from internal medicine. Doctors, nurses, and other healthcare workers are considered health role models and have the additional responsibility of educating the public about their health. Earlier intervention for substance use is important for decreasing the national health burden, and it is essential for HCPs to facilitate this. The role of education in highlighting the dangers of substance use to HCPs, leading to abuse and dependency, and how this may come to affect their patients remains invaluable especially in our setting.

## Supporting information

S1 Data This is the de-identified data of the study participants. (XLSX)
